# Prevalence of electrocardiographic abnormalities in primary care patients according to sex and age group. A retrospective observational study

**DOI:** 10.1590/1516-3180.2017.0222290817

**Published:** 2017-12-18

**Authors:** Julia Pereira Afonso dos Santos, Antonio Luiz Pinho Ribeiro, Diomildo Andrade-Junior, Milena Soriano Marcolino

**Affiliations:** I Medical Student, Department of Internal Medicine, Medical School, Universidade Federal de Minas Gerais, Belo Horizonte (MG), Brazil.; II MD, PhD. Full Professor, Department of Internal Medicine, Medical School, Universidade Federal de Minas Gerais, Belo Horizonte (MG), Brazil.; III MD. Physician, Department of Internal Medicine, Medical School, Universidade Federal de Minas Gerais, Belo Horizonte (MG), Brazil.; IV MD, MSc, PhD. Associate Professor, Department of Internal Medicine, Medical School, Universidade Federal de Minas Gerais, Belo Horizonte (MG), Brazil.

**Keywords:** Electrocardiography, Sex, Age groups, Primary health care

## Abstract

**BACKGROUND:**

Knowledge of the prevalence of electrocardiographic abnormalities in a population is useful for interpreting the findings. The aim here was to assess the prevalence of electrocardiographic abnormalities and self-reported comorbidities and cardiovascular risk factors according to sex and age group among Brazilian primary care patients.

**DESIGN AND SETTING:**

Observational retrospective study on consecutive primary care patients in 658 cities in the state of Minas Gerais, Brazil, whose digital electrocardiograms (ECGs) were sent for analysis to the team of the Telehealth Network of Minas Gerais (TNMG).

**METHODS:**

All ECGs analyzed by the TNMG team in 2011 were included. Clinical data were self-reported and electrocardiographic abnormalities were stratified according to sex and age group.

**RESULTS:**

A total of 264,324 patients underwent ECG examinations. Comorbidities and cardiovascular risk factors were more frequent among women, except for smoking. Atrial fibrillation and flutter, premature beats, intraventricular blocks, complete right bundle branch block and left ventricular hypertrophy were more frequent among men, and nonspecific ventricular repolarization abnormalities and complete left bundle branch block among women.

**CONCLUSION:**

Electrocardiographic abnormalities were relatively common findings, even in the younger age groups. The prevalence of electrocardiographic abnormalities increased with age and was higher among men in all age groups, although women had higher frequency of self-reported comorbidities.

## INTRODUCTION

Cardiovascular diseases are the leading cause of death worldwide^1^ and have a high socioeconomic impact.^2^ The high mortality and morbidity associated with these diseases makes diagnosis and management of these conditions essential in clinical practice.

Electrocardiograms (ECGs) are important examinations for assessing cardiac disease. Prior knowledge of the prevalence of abnormalities in the population studied is useful for interpreting ECG findings.^3^ Additionally, electrocardiographic abnormalities are independently associated with the incidence of coronary heart disease and with poor cardiac disease outcomes.^4^

The prevalence of electrocardiographic abnormalities varies with age and sex.^3^^,^^5^^,^^6^ Recently, many studies have focused on the unique aspects of cardiac disease in women, in order to optimize its diagnosis and treatment.^4^^,^^7^^,^^8^ In this regard, the present study may contribute to the literature on the subject through highlighting the differences in ECG findings between men and women in separate age groups, in a large sample of Brazilian primary care patients, and through discussing the particularities of female ECGs in relation to male ones.

## OBJECTIVE

The aim of this study was to analyze the prevalences of self-reported comorbidities and electrocardiographic abnormalities according to age and sex among Brazilian primary care patients. Some specific aspects of women’s ECGs in relation to men’s ones are also discussed.

## METHODS

This retrospective observational study included all ECGs that were recorded in primary care units and then analyzed by cardiologists of the Telehealth Network of Minas Gerais (TNMG), a Brazilian large-scale public telehealth service, from January 1 to December 31, 2011. During this period, the service provided support to primary care practitioners in 658 municipalities in the state of Minas Gerais, among which 85% have fewer than 14,000 inhabitants. It performed teleconsultations and remote interpretation of diagnostic tests, including ECG analysis.^9^

Digital 12-lead electrocardiograms were produced using tele-electrocardiograph machines made by Tecnologia Eletrônica Brasileira (TEB; São Paulo, Brazil) or Micromed Biotechnology (Brasília, Brazil) and were sent over the internet to an analysis center, from which the examinations were immediately forwarded to a team of cardiologists, who analyzed the ECGs using standardized criteria.^10^ The team of cardiologists was composed of ten cardiologists who had been trained and were experienced in ECG analysis. Their ECG analyses were also subject to periodic auditing and feedback.^9^ Only one individual reviewed each ECG.

The clinical data were self-reported and were gathered immediately before the patients were subjected to the ECG exam. A standard questionnaire was used, which sought data including age, sex, medications in use, comorbidities (hypertension, diabetes, obesity, dyslipidemia, chronic kidney disease, chronic obstructive pulmonary disease and coronary artery disease), prior acute myocardial infarction, smoking and family history of coronary heart disease.

For the purpose of this study, all consecutive ECGs from January 1, 2011, to December 31, 2011, were analyzed. Electrocardiograms with technical issues such as interference or errors in the placement of electrodes were excluded. The proportion of atrial flutter was considered along with the proportion of atrial fibrillation, as has also been done in other epidemiological studies.^11^ The prevalence of electrocardiographic abnormalities was evaluated and stratified according to sex and age groups. The age groups encompassed every two decades of life: from 0 to 19.9 years of age; 20 to 39.9; 40 to 59.9; 60 to 79.9; and greater than or equal to 80 years. Rankings of the most common abnormalities according to age group and sex were elaborated and a table of the final ranking according to sex and age group was constructed.

The IBM SPSS statistics software for Windows version 20.0 (2011 release; IBM Corporation, Armonk, NY, USA) was used for the statistical analyses. Categorical variables were reported as counts and percentages; continuous variables were reported as means and standard deviations (SD) or medians with interquartile range (IQR), as appropriate. This study was approved by the Research Ethics Committee of the Federal University of Minas Gerais.

## RESULTS

Over the course of this study, ECG recordings from 264,324 primary care patients were analyzed by the TNMG cardiology team; 58.7% of the patients were women. The patients’ mean age was 51 ± 19 years; 7.2% of them were between zero and 19.9 years of age; 21.3% between 20 and 39.9 years; 37.6% between 40 and 59.9 years; 28.2% between 60 and 79.9 years; and 5.0% greater than 80 years. In 0.7% of the examinations, the patient’s age was not included. The youngest group was excluded from further evaluation here.

Table 1 shows the prevalences of self-reported comorbidities. Tables 2A and 2B show the prevalences of electrocardiographic abnormalities according to sex and age groups. Table 3 shows the ranking of the prevalences of electrocardiographic abnormalities according to sex and age groups.

**Table 1:** Reported comorbidities and risk factors, according to sex and age group (n = 264,324)

Age group												
Comorbidity/risk factors	20-39.9 years			40-59.9 years			60-79.9 years			> 80 years		
	F (n= 35,463)	M (n = 20,922)	P	F (n = 61,911)	M (n = 37,555)	P	F (n = 42,501)	M (n = 31,973)	P	F (n = 7,596)	M (n = 5,685)	P
Hypertension	4,682 (13.20%)	2,549 (12.18%)	< 0.001	22,578 (36.47%)	11,364 (30.26%)	< 0.001	22,007 (51.78%)	13,917 (43.53%)	< 0.001	4,126 (54.32%)	2,677 (47.09%)	< 0.001
Chagas disease	490 (1.38%)	283 (1.35%)	0.774	2,346 (3.79%)	1,313 (3.50%)	0.017	1,755 (4.13%)	989 (3.09%)	< 0.001	252 (3.32%)	117 (2.06%)	< 0.001
Diabetes mellitus type 2	616 (1.74%)	282 (1.35%)	< 0.001	3,685 (5.95%)	1,625 (4.33%)	< 0.001	4,856 (11.43%)	2,082 (6.51%)	< 0.001	709 (9.33%)	304 (5.35%)	< 0.001
Dyslipidemia	370 (1.04%)	194 (0.93%)	0.181	2,104 (3.40%)	972 (2.59%)	< 0.001	2,284 (5.37%)	991 (3.10%)	< 0.001	309 (4.07%)	115 (2.02%)	< 0.001
Smoking	1,755 (4.95%)	904 (4.32%)	< 0.001	4,601 (7.43%)	4,699 (12.51%)	< 0.001	1,703 (4.01%)	3,255 (10.18%)	< 0.001	217 (2.86%)	430 (7.56%)	< 0.001
COPD	190 (0.54%)	67 (0.32%)	< 0.001	388 (0.63%)	186 (0.50%)	0.008	362 (0.85%)	337 (1.05%)	0.005	88 (1.16%)	93 (1.64%)	0.023
Chronic renal disease	159 (0.45%)	72 (0.34%)	0.061	331 (0.53%)	165 (0.44%)	0.039	227 (0.53%)	165 (0.52%)	0.758	31 (0.41%)	28 (0.49%)	0.512
History of myocardial infarction	105 (0.30%)	50 (0.24%)	0.211	379 (0.61%)	338 (0.90%)	< 0.001	430 (1.01%)	440 (1.38%)	< 0.001	71 (0.93%)	64 (1.13%)	0.295
Family history of coronary artery disease	5,547 (15.64%)	2,875 (13.74%)	< 0.001	10,394 (16.79%)	5,379 (14.32%)	< 0.001	6,780 (15.95%)	4,344 (13.59%)	< 0.001	1,079 (14.20%)	744 (13.09%)	0.067

F = female examinations; M = male examinations; P = P-value; COPD = chronic obstructive pulmonary disease.

**Table 2A:** Electrocardiograms abnormalities according to sex and age group: rhythm abnormalities, atrioventricular block and intraventricular conduction defects (n = 264,324)

Age group												
Abnormalities	20-39.9 years			40-59.9 years			60-79.9 years			> 80 years		
	F (n = 35,463)	M (n = 20,922)	P	F (n = 61,911)	M (n = 37,555)	P	F (n = 42,501)	M (n = 31,973)	P	F (n = 7,596)	M (n = 5,685)	P
Rhythm disorders												
Sinus rhythm	33,855 (95.46%)	19,736 (94.33%)	< 0.001	59,228 (95.66%)	35,325 (94.06%)	< 0.001	39,623 (93.2%)	28,978 (90.6%)	< 0.001	6,662 (87.7%)	4,757 (83.7%)	< 0.001
Ectopic atrial rhythm	84 (0.23%)	43 (0.20%)	0.448	98 (0.15)	76 (0.20)	0.107	73 (0.2%)	85 (0.3%)	0.006	23 (0.3%)	22 (0.4%)	0.450
Multifocal atrial rhythm	1 (0.002%)	1 (0.004%)	0.706	3 (0.0%)	4 (0.0%)	0.290	8 (0.0%)	9 (0.0%)	0.466	4 (0.1%)	3(0.1%)	1.000
Pacemaker	20 (0.05%)	24 (0.11%)	0.017	130 (0.20%)	106 (0.28%)	0.023	245 (0.6%)	185 (0.6%)	1.000	75 (1.0%)	78 (1.4%)	0.048
Junctional rhythm	18 (0.05%)	15 (0.07%)	0.321	40 (0.06%)	23 (0.06%)	0.838	46 (0.1%)	56 (0.2%)	0.016	18 (0.2%)	16 (0.3%)	0.611
Atrial fibrillation and flutter	49 (0.13%)	49 (0.23%)	0.008	336 (0.54%)	448 (1.19%)	< 0.001	1,188 (2.8%)	1,429 (4.5%)	< 0.001	527(6.9%)	586 (10.3%)	< 0.001
Supraventricular extrasystole and ventricular extrasystole	5 (0.014%)	2 (0.009%)	0.640	25 (0.04%)	20 (0.05%)	0.355	64 (0.2%)	71 (0.2%)	0.029	45 (0.6%)	49 (0.9%)	0.075
Supraventricular extrasystole	213 (0.60%)	145 (0.69%)	0.182	458 (0.73)	356 (0.94%)	< 0.001	1,109 (2.6%)	1,228 (3.8%)	< 0.001	505 (6.6%)	464 (8.2%)	0.001
Ventricular extrasystole	253 (0.71%)	148 (0.70%)	0.934	897 (1.44%)	668 (1.77%)	< 0.001	1,489 (3.5%)	1,589 (5.0%)	< 0.001	480 (6.4%)	521 (9.2%)	< 0.001
Atrioventricular (AV) blocks												
AV block 1	185 (0.52%)	183 (0.87%)	< 0.001	533 (0.86%)	627 (1.66%)	< 0.001	939 (2.2%)	1,257 (3.9%)	< 0.001	396 (5.2%)	457 (8.0%)	< 0.001
AV 2:1 advanced	6 (0.0169%)	8 (0.038%)	0.121	15 (0.02%)	35 (0.09%)	< 0.001	36 (0.1%)	45 (0.1%)	0.024	20 (0.3%)	20 (0.4%)	0.425
Complete AV block	5 (0.014%)	7 (0.03%)	0.128	16 (0.02%)	16 (0.04%)	0.153	33 (0.1%)	28 (0.1%)	0.700	17 (0.2%)	22 (0.4%)	0.104
AV block 2	6 (0.0169%)	7 (0.03%)	0.211	12 (0.019%)	29 (0.07%)	< 0.001	28 (0.1%)	36 (0.1%)	0.042	16 (0.2%)	15 (0.3%)	0.590
AV block 2:1	0	0		3 (0.004%)	8 90.02%)	0.017	10 (0.0%)	9 (0.0%)	0.818	4 (0.1%)	6 (0.1%)	.343
Intraventricular blocks												
Left anterior hemiblock	370 (1.04%)	502 (2.39%)	< 0.001	2,198 (3.55%)	2,542 (6.76%)	< 0.001	3,566 (8.4%)	4,545 (14.2%)	< 0.001	1,004 (13.2%)	1,144 (20.1%)	< 0.001
Left posterior hemiblock	139 (0.39%)	187 (0.89%)	< 0.001	114 (0.18%)	154 (0.41%)	< 0.001	103 (0.2%)	118 (0.4%)	0.002	22 (0.3%)	16 (0.3%)	0.997
Complete right bundle branch block	296 (0.83%)	331 (1.58%)	< 0.001	1,360 (2.19%)	1,258 (3.34%)	< 0.001	1,804 (4.2%)	1,982 (6.2%)	< 0.001	474 (6.2%)	558 (9.8%)	< 0.001
Complete left bundle branch block	46 (0.129%)	38 (0.18%9)	0.123	580 (0.93%)	3,389 (0.90%)	0.556	1,408 (3.3%)	935 (2.9%)	0.003	480 (6.32%)	317 (5.58%)	0.077

F = female examinations; M = male examinations; P = P-value.

**Table 2B:** Electrocardiograms abnormalities according to sex and age group: enlargement and hypertrophy, ischemia and other abnormalities (n = 264,324)

Age group												
Abnormalities	20-39.9 years			40-59.9 years			60-79.9 years			> 80 years		
	F (n = 35,463)	M (n = 20,922)	P	F (n = 61,911)	M (n = 37,555)	P	F (n = 42,501)	M (n = 31,973)	P	F (n = 7,596)	M (n = 5,685)	P
Enlargement and hypertrophy												
Right atrial enlargement	38 (0.10%)	28 (0.13%)	0.371	81 (0.13%)	82 (0.21%)	0.001	103 (0.2%)	86 (0.3%)	0.509	16 (0.2%)	16 (0.3%)	0.477
Right ventricular enlargement	11 (0.03%)	30 (0.14%)	< 0.001	16 (0.02%)	36 (0.09%)	< 0.001	32 (0.1%)	45 (0.1%)	0.007	11 (0.1%)	10 (0.2%)	0.665
Left atrial hypertrophy	218 (0.61%)	238 (1.13%)	< 0.001	1,157 (1.86%)	1,245 (3.31%)	< 0.001	1,470 (3.5%)	1,785 (5.6%)	< 0.001	357 (4.7%)	308 (5.4%)	0.064
Left ventricular hypertrophy	152 (0.42%)	412 (1.96%)	< 0.001	1,055 (1.70%)	1,526 (4.06%)	< 0.001	2,036 (4.8%)	2,250 (7.0%)	< 0.001	658 (8.7%)	521 (9.2%)	0.324
Ischemia												
Subendocardial	16 (0.04%)	13 (0.06%)	0.278	135 (0.21%)	6 (0.25%)	0.233	233 (0.5%)	141 (0.4%)	0.042	79 (1.0%)	40 (0.7%)	0.051
Subepicardial	37 (0.10%)	51 (0.24%)	< 0.001	273 (0.44%)	276 (0.73%)	< 0.001	390 (0.9%)	333 (1.0%)	0.089	80 (1.1%)	51 (0.9%)	0.376
Q wave	10 (0.02%)	14 (0.06%)	0.031	136 (0.21%)	194 (0.51%)	< 0.001	189 (0.4%)	298 (0.9%)	< 0.001	51 (0.7%)	56 (1.0%)	0.049
Poor R wave progression	195 (0.549%)	286 (1.366%)	< 0.001	829 (1.33%)	922 (2.45%)	< 0.001	1,279 (3.0%)	1,642 (5.1%)	< 0.001	354 (4.7%)	381 (6.7%)	< 0.001
Other abnormalities												
ST segment elevation	79 (0.22%)	490 (2.34%)	< 0.001	136 (0.21%)	537 (1.42%)	< 0.001	120 (0.3%)	262 (0.8%)	< 0.001	36 (0.5%)	42 (0.7%)	0.051
ST segment depression	44 (0.12%)	27 (0.12%)	0.872	304 (0.49%)	165 (0.43%)	0.249	382 (0.9%)	278 (0.9%)	0.692	114 (1.5%)	53 (0.9%)	0.004
Peaked T waves	3 (0.008%)	9 (0.043%)	0.007	9 (0.01%)	24 (0.06%)	< 0.001	5 (0.0%)	26 (0.1%)	< 0.001	7 (0.1%)	7 (0.1%)	0.600
Long QT	7 (0.019%)	3 (0.01%)	0.642	21 (0.03%)	12 (0.03%)	0.869	26 (0.1%)	15 (0.0%)	0.433	5 (0.1%)	6 (0.1%)	0.546
WPWS	86 (0.24%)	66 (0.31%)	0.107	95 (0.15%)	79 (0.21)	0.037	48 (0.1%)	51 (0.2%)	0.103	9 (0.1%)	4 (0.1%)	0.418
Brugada pattern	5 (0.014%)	8 (0.038%)	0.068	4 (0.006%)	13 (0.034%)	0.001	4 (0.0%)	7 (0.0%)	0.224	0	0	
Low QRS	39 (0.109%)	13 (0.062%)	0.071	152 (0.24%)	83 (0.22%)	0.440	132 (0.3%)	139 (0.4%)	0.006	40 (0.5%)	29 (0.5%)	0.998
Early repolarization	112 (0.31%)	851 (4.06%)	< 0.001	106 (0.17%)	710 (1.89%)	< 0.001	78 (0.2%)	287 (0.9%)	< 0.001	6 (0.1%)	27 (0.5%)	< 0.001
Nonspecific ventricular repolarization abnormalities	3,278 (9.24%)	2,152 (10.28%)	< 0.001	12,578 (20.3%)	6,919 (18.42%)	< 0.001	12,994 (30.6%)	8,784 (27.5%)	< 0.001	2,890 (38.0%)	2,035 (35.8%)	0.008
Normal	28,599 (80.64%)	14,787 (70.67%)	< 0.001	40,945 (66.13%)	22,481 (59.86%)	< 0.001	19,843 (46.7%)	13,031 (40.8%)	< 0.001	2,232 (29.4%)	1,378 (24.2%)	< 0.001

F = female examinations; M = male examinations; P = P-value; WPWS = Wolff-Parkinson-White syndrome.

**Table 3:** Ranking of electrocardiograms abnormalities according to sex and age group (n = 264,324)

Age group								
Abnormalities	20-39.9 years		40-59.9 years		60-79.9 years		≥ 80 years	
	F (n = 35,463)	M (n = 20,922)	F (n = 61,911)	M (n = 37,555)	F (n = 42,501)	M (n = 31,973)	F (n = 7,596)	M (n = 5,685)
Rhythm disorders								
Ectopic atrial rhythm	13	16	21	22	22	22	20	21
Multifocal atrial rhythm	33	33	33	34	32	33	33	33
Pacemaker	21	21	18	18	14	17	15	12
Junctional rhythm	22	22	24	27	25	24	23	25
Atrial fibrillation and flutter	15	15	11	12	9	8	4	3
Supraventricular extrasystole and ventricular extrasystole	29	32	25	28	23	23	17	16
Supraventricular extrasystole	6	12	10	13	10	10	5	7
Ventricular extrasystole	4	11	6	9	5	7	7	6
Atrioventricular (AV) blocks								
AV block 1	8	10	9	10	11	9	9	8
AV block 2:1 advanced	27	27	29	24	26	26	22	23
Complete AV block	30	30	27	29	27	29	24	22
AV block 2	28	29	30	25	29	28	26	27
AV block 2:1	34	34	34	32	31	32	32	31
Intraventricular blocks								
Left anterior hemiblock	2	3	2	3	2	2	2	2
Left posterior hemiblock	10	9	19	17	19	20	21	24
Complete right bundle branch block	3	6	3	5	4	4	8	4
Complete left bundle branch block	16	17	8	2	7	11	6	10
Enlargement and hypertrophy								
Right atrial enlargement	19	19	23	20	20	21	25	26
Right ventricular enlargement	24	18	28	23	28	27	27	28
Left atrial hypertrophy	5	8	4	6	6	5	10	11
Left ventricular hypertrophy	9	5	5	4	3	3	3	5
Ischemia								
Subendocardial	23	24	17	33	15	18	14	18
Subepicardial	20	14	13	14	12	12	13	15
Q wave	25	23	15	15	16	13	16	13
Poor R wave progression	7	7	7	7	8	6	11	9
Other abnormalities								
ST segment elevation	14	4	16	11	18	16	19	17
St segment depression	17	20	12	16	13	15	12	14
Peaked T waves	32	26	31	26	33	30	29	29
Long QT	26	31	26	31	30	31	31	30
WPWS	12	13	22	21	24	25	28	32
Brugada	31	28	32	30	34	34	34	34
Low QRS	18	25	14	19	17	19	18	19
Early repolarization	11	2	20	8	21	14	30	20
Nonspecific changes of ventricular repolarization	1	1	1	1	1	1	1	1

F = female examinations; M = male examinations; WPWS = Wolff-Parkinson-White syndrome.

Hypertension was the most frequent comorbidity, except in the group from 20 to 39.9 years of age, followed by a family history of coronary artery disease and smoking. In the group from 20 to 39.9 years of age, a family history of coronary artery disease was the most frequent risk factor for cardiovascular disease. From the age of 60 years, diabetes mellitus began to show significant prevalence: 11.4% and 6.5% respectively among men and women between 60 and 79.9 years of age and 9.3% and 5.3% among those aged 80 years and over. In general, the prevalence of comorbidities was higher in women of all age groups. The most common electrocardiographic abnormalities of all were nonspecific ventricular repolarization abnormalities, with prevalences ranging from 9.2% in women aged 20 to 39.9 years to 38.0% in those aged 80 and over (P = 0.008).

In the age group from 20 to 39.9 years, 80.6% of the tests in males and 70.7% in females were normal. The main electrocardiographic abnormality in women was left anterior hemiblock (LAH)^12^ (1.0%), followed by complete right bundle branch block (RBBB) (0.8%). In men, early repolarization pattern (ERP) (4.1%) and LAH (2.4%) were the most prevalent.

Between 40 and 59.9 years of age, 66.1% and 59.9% of the examinations among women and men respectively were normal. Among women, the most common abnormalities remained similar to those of the younger age group described above, despite increases in their prevalence (3.6% for LAH and 2.2% for RBBB). Among men, these findings became predominant (6.8% and 3.3%, respectively) and the prevalences of left atrium enlargement and ventricle hypertrophy increased (3.3% and 4.1%, respectively).

In the age group from 60 to 79.9 years, 46.7% of females and 40.8% of males presented normal results from the tests. Left ventricular hypertrophy became the second most prevalent abnormal result, following LAH (4.8% in women, 7.0% in men). Left bundle branch block (LBBB) (3.3% and 2.9%, respectively), first-degree atrioventricular block (AVB) (2.2% and 3.9%) and atrial fibrillation and flutter (2.8% and 4.5%) became more frequent.

In patients aged greater than or equal to 80 years, 70.6% of the women and 75.8% of the men showed abnormalities on the electrocardiogram. In both sexes, there was significantly increased prevalence of atrial fibrillation and flutter, especially among men (10.3%). In women, left ventricular hypertrophy remained a major result (8.7%), as did RBBB (6.2%), LBBB (6.3%) and LAH (13.2%). LAH was present in over 20% of examinations on males and first-degree AVB in 8.0%.

## DISCUSSION

In this study, on a large sample of primary care patients, electrocardiographic abnormalities were relatively common findings, even in the younger age groups. In the age group from 20 to 39.9 years, 19.4% of the women and 29.3% of the men had at least one abnormal result. The prevalence of abnormalities increased with age and was higher among males in all age groups. Atrial fibrillation and flutter, premature beats, intraventricular block, complete right bundle branch block and left ventricle hypertrophy were more frequent among men. Women had higher prevalences of nonspecific ventricular repolarization abnormalities and complete left bundle branch block.

Most examinations (87.1%) were conducted on patients aged between 20 and 79.9 years. Women presented a higher proportion of self-reported comorbidities, except for smoking. This reinforces the findings in the literature on this subject, which indicate that women care more about their health and therefore tend to be more aware of their medical conditions.^12^^,^^13^

With regard to comorbidities and cardiovascular risk factors, hypertension was the most common one (34.2% and 28.9% in women and men, respectively) from 20 years of age onwards, followed by family history of coronary artery disease (16.0% and 13.6% in women and men). The prevalence of hypertension in the population aged 60-79.9 years in the present analysis (48.2%) was similar to what was found among subjects from 60 to 70 years of age (48.6%) in a cross-sectional study that investigated hypertension in the population of a Brazilian state capital.^14^ In another study, in which household surveys were conducted in 15 Brazilian state capitals and in the federal district, the prevalence of self-reported hypertension among individuals aged 25-39 years (7.4% to 15.7%) was similar to what was found in the present study in the age group of 20-40 years.^15^ The Brazilian Longitudinal Study of Adult Health (ELSA-Brasil) also had similar figures.^16^ This suggests that our sample may be representative of the Brazilian population.

Sex differences regarding hypertension are well known, from epidemiology to pathophysiology to target organ damage. Women have higher awareness, treatment and control rates and lower prevalence of left ventricular hypertrophy (LVH).^7^ This was seen in the sample of the present study: while reports of disease were higher in females, , males had higher prevalence of LVH in all age groups.

Self-reported diabetes was more frequent among females, mostly in individuals over 60 years of age. In the literature, slightly higher prevalence of diabetes in males has been reported worldwide. Nonetheless, studies from the Caribbean and from southern Africa showed higher prevalence of diabetes in women than in men, which was a pattern similar to the one found in the present study. This was possibly due to higher rates of obesity among females from such developing regions, since obesity is one of the greatest risk factors for diabetes.^17^

There were fewer smokers aged between 20 and 39.9 years than in the age groups of 40-59.9 and 60-79.9 years. This corroborates the results from several studies that have demonstrated reductions in smoking rates over recent decades, mainly influenced by tobacco control initiatives such as tax increases on these products and creation of restrictions on public smoking, among other equally effective measures.

Octogenarians reported lower frequency of Chagas disease, diabetes mellitus, smoking and dyslipidemia than did younger subjects, thus indicating that people who reach older age groups usually have fewer comorbidities and cardiovascular risk factors, which may be related to survival bias.

The prevalence of chronic kidney disease (CKD) is very likely to be underestimated: about 0.5% among women and men over 60 years. A study in Juiz de Fora, a city in the same Brazilian state, showed that the prevalence in the same age group was 25.2%. It is possible that many patients were not aware of their condition, which thus emphasizes the need for screening, especially among individuals with high blood pressure and diabetes, which are the leading risk factors for CKD.^18^

Differences between the sexes regarding the cardiovascular system result from differences in gene expression from the sex chromosomes. This can also be further modified through the influence of sex-related hormones and other environmental factors, thereby resulting in sex-specific gene expression.^8^ Thus, electrocardiographic abnormalities may show primary differences between men and women. In the present study, 33.9% of the women and 40.1% of the men aged 40-59.9 years presented abnormal examinations. This was similar to the findings of another Brazilian study that also evaluated such abnormalities stratified by age, although this other study did not examine the prevalence in relation to sex and also included patients from secondary care.^19^

LAH was one of the most common disorders in all age groups, with increasing prevalence according to age. It may be caused by hypertension, cardiomyopathies, Chagas disease in endemic countries and Lev and Lenegre disease, and may form part of a benign senile degenerative process.^20^ However, this abnormality has little or no correlation with poor prognosis and is poorly associated with higher numbers of comorbidities.^20^ The prevalence rates for LAH in the combined population aged 40-79.9 years were 5.5% for women and 10.2% for men. This was compatible with several studies that have indicated that the prevalences of left axis deviation (which could be an indicator of LAH) and of LAH among men are around twice as high as among women.^3^ One example of such findings comes from an Indian study in which different rates of abnormal ECG results between the sexes were observed among people aged 45-74 years: 5.7% for women and 9.6% for men. There was also strong agreement regarding the prevalence of left ventricular hypertrophy between this Indian study and the present study: 2.9% and 5.1% in the present study, versus 2.8% and 4.6% in the Indian study, in women and men respectively.^6^

The prevalence of atrial fibrillation was strongly associated with greater age, and it was higher in men than in women, in all age groups. Our findings regarding the prevalence of atrial fibrillation according to age and sex were similar to data from high-income countries.^11^ This confirms and extends the findings of a previous paper from our group,^21^ from a subsample of the data used in the present study that was analyzed without the Minnesota Code. Since atrial fibrillation is a major risk factor for stroke, but there is no national health policy to promote primary and secondary stroke prevention among patients with atrial fibrillation (the new oral anticoagulants are not provided through the public health system and there are not enough anticoagulation clinics to control patients on warfarin),^22^ the data provided by the present study is very important for stakeholders.

Another very frequent finding in all age groups was RBBB, which gives rise to a threefold increased risk of cardiovascular events and has been correlated with larger numbers of comorbidities.^23^ RBBB also presented increasing prevalence with age, as had already been observed in the evaluation on RBBB within the Copenhagen City Heart Study.^24^ Complete RBBB had higher prevalence in the present study than in the Danish study (4.0% and 2.5% in men and women respectively, versus 1.5% and 0.5%).^24^ One hypothesis that would explain this discrepancy is the higher number of patients with Chagas disease in Brazil.

It has been well established that men present higher frequencies of intraventricular block and RBBB than do women.^25^ This was also found in the present study in relation to LAH, left posterior hemiblock and RBBB, but not in relation to LBBB. A statistically significant difference in the frequency of LBBB between men and women was only present in the age group from 60 to 79.9 years, which is understandable, given the usually late onset of LBBB.^26^ In this group, the prevalence was 2.9% in men and 3.3% in women. Other studies have also found similar prevalences of LBBB in both sexes^3^^,^^26^ but none of them further explored the slightly higher prevalence of LBBB among women.

Nonspecific ventricular repolarization abnormalities were the most prevalent abnormalities in all age groups. This is consistent with the previously mentioned American study that evaluated electrocardiographic disorders in 20,962 people according to sex and age.^5^ These abnormalities have been correlated with significantly higher risk of fatal coronary heart disease,^27^ for which primary arrhythmia is the main mechanism.^28^ This ECG disorder was more prevalent among women, and this might be explained by the significant influence of sex hormones on the QT interval in women: whereas this component is only shortened through the influence of testosterone in men, significant estrogen activity in women prolongs this interval while their progesterone acts similarly to testosterone.^29^ These nonspecific repolarization abnormalities were also found to be predictors of CHD events and CHD death among postmenopausal women.^30^

Chagas disease is still highly prevalent in Brazil. Out of the 5.7 million people chronically infected in Latin America, 20% are in this country.^31^ The most common electrocardiographic findings in Chagas disease are RBBB (22.7%) and LAH (22.5%). In addition to these, second and third-degree atrioventricular blocks and atrial fibrillation are also strongly associated with Chagas disease.^32^ In the present study, 2.9% of the patients reported having Chagas disease and, as previously described, this may explain the higher prevalence of RBBB in relation to other studies.^27^

Left ventricular hypertrophy (LVH) is an independent predictor of morbidity and cardiovascular mortality and tends to increase with age.^33^ The risk is particularly increased when associated with ventricular repolarization abnormalities.^34^ The main etiologies of left ventricle hypertrophy are hypertension, hypertrophic cardiomyopathy and dilated cardiomyopathy, coronary artery disease, valvular disease, obesity, diabetes mellitus, drug abuse and chronic kidney disease.^35^ In the present study, although the prevalence of hypertension was similar to that of other studies, as already mentioned, left ventricular hypertrophy remained below 10%, even in older individuals: 1.7% in women and 4.0% in men aged 40 to 59; 4.8% and 7.0% respectively between the ages of 60 and 79 years; and 8.7% and 9.2% among individuals aged 80 years and over. One hypothesis to explain this discrepancy is the low sensitivity of electrocardiograms for detecting this abnormality, in comparison with echocardiograms.^33^

Interestingly, ECG abnormalities suggestive of acute ischemia, i.e. signs of subendocardial and subepicardial injury, were 0.3% and 0.6% overall, even though the present study was on tests performed within primary care. These cases are supposed to be attended in emergency centers. However, many of the municipalities studied here do not have any emergency units or hospitals, and therefore patients seek care for emergency conditions at primary care centers. In addition, many patients become so used to attending primary care centers that they seek help there even in emergency situations.

This study has certain limitations. The comorbidities and medications were self-reported, so they may have been underreported. The electrocardiographic reports followed predetermined patterns, using criteria established by the Brazilian Society of Cardiology.^10^ These criteria have not yet been validated in as many population-based studies as the Minnesota code.^36^ However, the criteria used reflect current practices in Brazil, thus ensuring the ability to generalize the results to other primary care settings in this country.

## CONCLUSION

This study on a large sample of primary care patients showed that electrocardiographic abnormalities were relatively common findings, even in the younger age groups. The prevalence of abnormalities increased with age and was higher in men in all age groups, even though women had higher frequency of self-reported comorbidities. Atrial fibrillation and flutter, premature beats, intraventricular blocks, complete right bundle branch block and left ventricle hypertrophy were more frequent in men. Women had higher prevalence of nonspecific ventricular repolarization abnormalities and complete left bundle branch block.

The correlations of age and sex with electrocardiographic abnormalities that were made through the present study may help towards increasing the predictive value of ECGs and contribute towards diagnosing and subsequently managing many common cardiovascular diseases within primary care. Furthermore, the findings from this study reinforce the importance of consolidating programs for prevention and screening of diseases that enhance cardiovascular risk such as hypertension, diabetes, hyperlipidemia and smoking.
